# A multi-dataset exploratory framework for understanding digital behavior and substance use risk profiles

**DOI:** 10.3389/fpubh.2026.1771271

**Published:** 2026-03-18

**Authors:** Perla Shiva Sindhu, Modigari Narendra

**Affiliations:** School of Computer Science and Engineering, Vellore Institute of Technology, Chennai, India

**Keywords:** behavioral triggers, digital environments, intervention strategies, peer pressure, predictive modeling, social influence, substance use

## Abstract

**Introduction:**

Substance use continues to evolve as a multidimensional public health challenge influenced by traditional behavioral triggers and emerging digital interactions. This study investigates how demographic factors, psychological states, and patterns of digital engagement shape substance use behaviors using multiple behavioral data sources.

**Methods:**

Quantitative analyses were conducted using the NHANES dataset and a Kaggle social media psychology dataset to identify statistical relationships and train predictive machine learning models for substance use indicators and digital behavioral patterns. Random Forest, XGBoost, AdaBoost, Support Vector Regression (SVR), and Logistic Regression models were evaluated, with hyperparameter tuning applied to improve predictive performance. In addition, a supplementary survey (*N* = 236) was collected and used as a qualitative interpretive layer to contextualize the relationship between digital behavior and substance use risk.

**Results:**

The analysis revealed nonlinear relationships between social media engagement, anxiety, and loneliness. Contrary to the widely cited linear dose–response assumption, anxiety scores plateaued at higher levels of digital engagement, suggesting that the qualitative nature of online interactions may exert greater influence on psychological distress than usage duration alone. Machine learning models demonstrated improved predictive performance after hyperparameter tuning across both datasets.

**Discussion:**

These findings highlight the importance of considering digital engagement patterns alongside traditional behavioral and demographic factors in substance use research. The results support the development of platform-specific digital well-being strategies, nuanced behavioral modeling approaches, and culturally sensitive interventions that integrate both objective behavioral data and subjective user experiences. The proposed multi-source evidence framework provides a foundation for future exploratory behavioral risk profiling and prevention systems.

## 1 Introduction

The substance usage continues to remain a major public health challenge with great implications in morbidity, mortality, and social harm across populations worldwide. While traditional research has concentrated on behavioral triggers such as stress, peer pressure, and emotional distress recent societal shifts driven by digital technologies have given rise to new and less understood factors or influences on substance usage behaviors that result from recent shifts in society. Social media sites, online peer networks, and digitally mediated social interactions increasingly shape adolescents’ and young adults’ attitudes and norms, besides coping strategies on substance use (21). The rapid expansion of the digital footprint has changed the nature of social influence, with exposure to substance related content, peer normalization, and online reinforcement systems extending beyond face to face interaction convened in conventional peer pressure. In contrast to the traditional peer pressure, digital environments allow persistent, large-scale, and algorithmically mediated interactions that can amplify or change behavioral risk factors. Existing studies on substance use have examined psychological, demographic, or behavioral factors in isolation, while treating digital engagement as a peripheral or secondary influence rather than as an integrated component of risk. Consequently, the combined and interactive effects of digital behavior with established psychological and demographic determinants remain insufficiently explored.

Recent studies have also investigated connection between social media and mental health outcomes such as anxiety and loneliness, but until now, the results remain contradictory and often overly simplified into linear relationships between exposure to online activity and negative psychosocial outcomes (22). Moreover, most studies rely on single source datasets or self-report instruments; hence, they fail to accommodate the complex non-linear interplay involving demographic characteristics, psychological states, digital engagement patterns, and substance use behaviors. Cultural and contextual variability further complicates this scenario, given the great differences among populations in social norms and digital practices. To close that gap, the present study complements a multivariate statistical analysis on social media related psychological indicators and self reported contextual experiences. Statistical and machine-learning methods were applied to the public NHANES and social media psychological datasets to identify key predictors of substance use and digital behavioral risks, consistent with recent computational approaches that analyze behavioral signals and linguistic patterns in online environments (24). A personalized survey will provide insights that are qualitative and contextual into stress, coping mechanisms, and perceived digital influences-again enabling interpretations of patterns from secondary datasets.

The primary objectives of this research were: (i) to investigate how demographic factors, psychological states, and digital engagement patterns individually and jointly relate to indicators of substance use risk; (ii) to gauge the predictive capacity of machine-learning models in identifying individuals exposed to elevated behavioral risk; and (iii) to place quantitative findings within a self-reported context to inform culturally appropriate and digitally informed preventive strategies. Merging behavioral science perspectives into data modeling will offer more subtle understandings of substance use in the digital age and provide empirically grounded frameworks for future risk assessment and intervention protocols relying on AI. The present framework predicts relative behavioral risk profiles within each dataset, defined according to dataset-specific substance use indicators (e.g., alcohol frequency, binge episodes, tobacco use, digital addiction scores). It does not predict clinical diagnoses or distinguish experimental use from substance use disorder severity. The term “risk” is therefore used in a probabilistic and exploratory sense.

## 2 Related work

Amaro et al. (1) studied at the link between addiction history and the individual’s proclivity toward habitual behavior, and specifically how neural mechanisms are pertained to this behavior formation. The paper discusses the application of Hidden Markov Models (HMM) to model habitual behavior with an 82% accuracy rate, plus Functional MRI analysis (75%) to assess neural activity. This paper will help further understand habitual behaviors in the context of more serious addiction. However, the study is inclined toward the workplace environment of addiction and does not explore wider demographic contexts. Moreover, the longitudinal effects of addiction are one area not explored in detail, tightening the study’s scope. Pomrenze et al. (2) focused on the effects of excessive social media use on work performance and the behavioral mechanisms leading to this addiction. Behavioral triggers such as peer pressure and job stress have been identified and examined for their impact on work-related social media involvement. Decision Trees (80%) and Regression Analysis (78%) are employed to segment behaviors and predict work performance. However, the most important limitation of this paper is that it asks the neat question about the workplace environment, thus requiring further examination of the greater social implications of social media addiction among several contexts and demographics.

Belfiore et al. (3) examines psycho-behavioral measures and the antecedents of addiction to social media, hence looking at the psychological implications of stopping social media use. The article claims that stopping social media use will raise stress and withdrawal-like behavioral symptoms. Support Vector Machines are applied for psycho-behavioral analyses, with an accuracy of 83%. However, while the study has clearly brought to the fore many insights concerning social media addiction, it is restricted, in that it emphasizes only the psychological effects ignoring an integrated approach with substance use disorders and other addictions. The spread of unethical behavior like cheating through online social networks and how peer behavior has influenced individual choices are explored by Nawi et al. (4). Agent-Based Modeling and Graph Neural Networks are then used to simulate this contagion process, achieving 85 and 80% accuracy, respectively and shows that social networks facilitate the propagation of behaviors but does not cover any possibilities for contagion of positive behaviors. Additionally, there is a need to further validate these models using real life data and explore behavioral contagion in contexts other than social media. On an examination of internet addiction neural dynamics, McKim et al. (5) examines and compares with that seen in brain activity patterns across addiction with regard to substances. Brain oscillations and dynamic neural activity are analyzed with Convolutional Neural Networks (CNNs) (87%) and Recurrent Neural Networks (RNNs) (83%). Although it is very useful in the mechanisms of internet addiction, this research is mostly concerned with internet addiction and lacks comparison with other forms of addiction like that of substances or gaming.

Sun et al. (6) developed a theoretical model based upon social cognitive theory intended to support health behavior change intervention in emerging research. State-space modeling (80%) and system-identification methods (78%) are applied in optimizing behavioral intervention using empirical testing. The current study do not have empirical evidence of its functioning because it is focusing on certain behaviors, such as substance use. More research has to be done on complete real-life applications of this model toward further development of understanding treatments against addictive behavior. Ahmed et al. (7) investigated the conduct relating to the activity brought about due to game engagement and places that alongside behavior related to addictions specifically that of substance use. Reinforcement Learning (RL) (85%) was the means through which engagement patterns and behavioral clustering (78%) were modeled. This study concludes that gaming addiction shares quite a few behavioral characteristics with substance addiction. However, this study only centers on gaming and does not address addiction in other areas, such as substances and social media. Cross-addiction patterns can be one of the important aspects in research to carry out. An agent-based model (88%) and social network analysis (82%) is used by Woo et al. (8) to simulate the spreading of opioids through social networks. It emphasizes the role of social structures in affecting the dynamics of opioid addiction. Yet, this study remains to go through the peer-review process, being a preprint; therefore, it requires future validation with real-world data. The narrow scope of the study on opioids considerably restricts its generalizability to other forms of substance use.

Tseng et al. (9) examines the socially toxic environments and racism as stressor aspects pertaining to substance use. Vulnerability to substance use disorders and a definition of a role for social aspects in addiction have been identified concerning the study. Although providing insights, the study lacks focus on a few interventions to address social inequalities. Recommendations for further research involve examining strategies to counteract the negative impact of toxic environments and racial stressors on addiction. Martín et al. (10) deals with the negative social interactions on drug-seeking behavioral patterns and social environments’ contributions to addiction and relapse. The paper shows that negative social influences are significant driving factors in the use of substances, with the help of recurrent neural networks (85% accuracy) in behavior prediction and sentiment analysis (72% accuracy) for interaction context analysis. With regards to this, the study lacks an explicit model to measure the actual impact of these forces and to apply it to the scenario of real-life addiction treatment context.

Khalid et al. (11) analyzed the biological, social, and psychological determinants of substance use disorder and co-occurring mental health conditions at diverse levels. These disorders’ co-occurrence predictions were modeled using hierarchical modeling (88%) and Bayesian networks (80%). However, the study falters in placing the predictive model within an integrated framework incorporating longer-term consequences of these co-occurring disorders and potential treatments for chronic sufferers battling both mental health and substance use problems. In the systematic review from Queen et al. (12), risk factors for adolescent drug abuse such as peer pressure and early onset of mental health problems are identified, along with family support and community participation as protective factors. Digital peer pressure is a newly emerging risk factor identified, thus the need for interventions to be culturally sensitive. The study used random forests (82%) for risk factor identification, whilst natural language processing (77%) for contextual factor extraction. However, from this review, it is obvious some emerging risks and studies regarding specific cultural contexts require further investigation.

Together, most of the research suggests that the AI driven behavior model holds a promise to detect risks associated with addiction and many studies have largely failed to translate the model into an intervention that is genuinely practical and deployable (23). Existing work frequently ignores cultural variability, heavily relies on single-source datasets, and rarely takes into account long term behavioral patterns or contextual psychological factors. Furthermore, it does not docus much on the challenges of model generalizability, population diversity, and data privacy. Conversely, this study addresses these challenges with a knowledge generating strategic plan of multi-source analysis consisting of the independent examination of NHANES and social media affected psychological datasets through statistical and machine-learning models, while a personalized survey offers cultural and behavioral context. It integrates demographic, psychological, and digital engagement indicators to fill gaps in the previous literature and offers a richer understanding of cross domain behavioral triggers related to substance use.

## 3 Methodology

**Figure 1:**
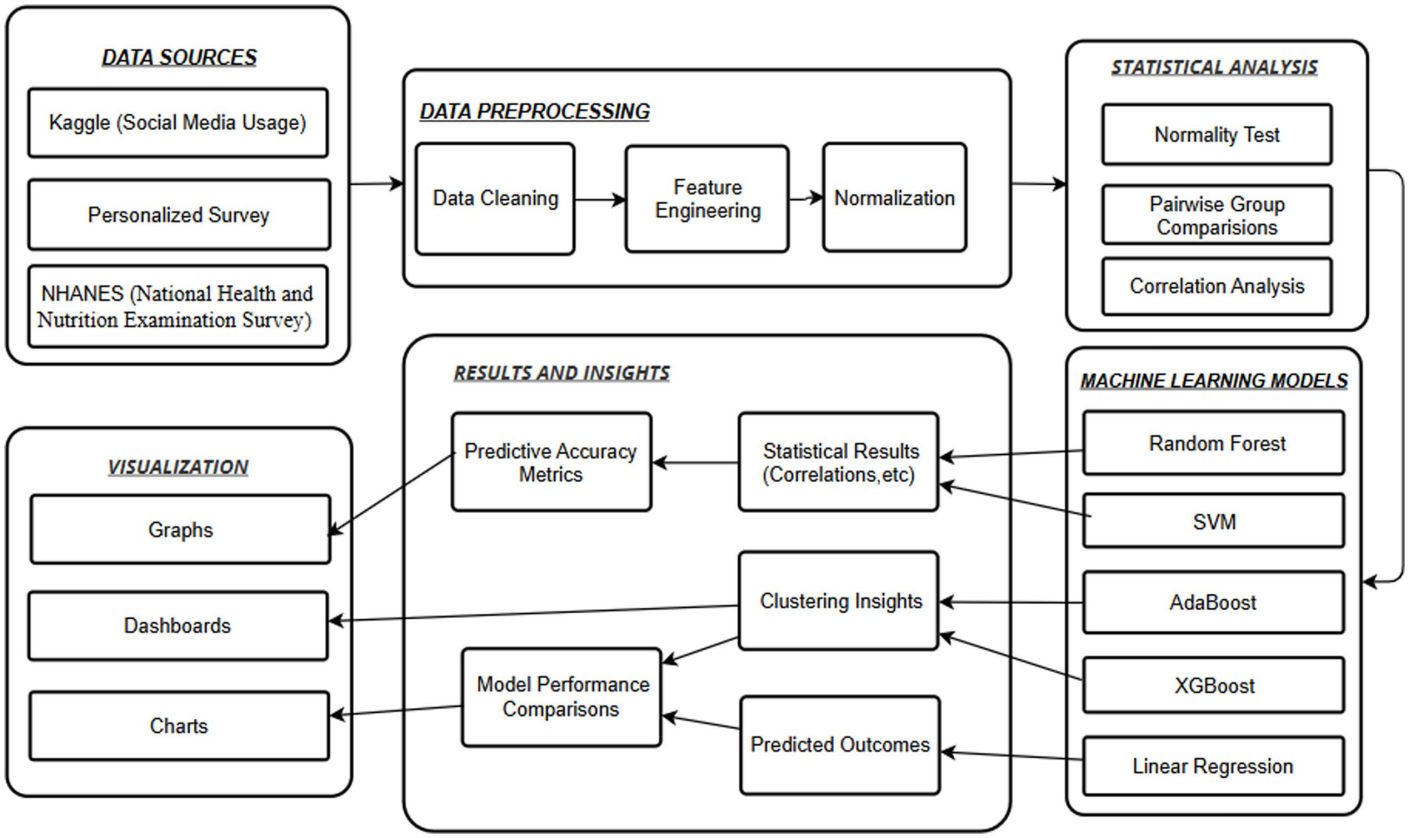
Flow diagram of the proposed methodology.

This section outlines the methodological framework for investigating behavioral triggers and social influences on substance usage patterns, focusing on digital engagement. This study does independent processing, analysis, and modeling of three independent datasets: NHANES, a Kaggle Social Media dataset, and a Personalized Survey dataset to examine the demographic, psychological, and digital factors related to substance use. Our methodology primary utilizes an information-driven paradigm based on large-scale behavioral data. While this framework is grounded in objective datasets to minimize the inherent biases of self-reporting, a supplementary survey (*N* = 236) was utilized strictly as a qualitative interpretive layer to validate the digital-substance interface in a specific social context. The proposed framework does not integrate datasets at the individual level nor generate a composite risk score across sources. Instead, it standardizes analytical procedures across heterogeneous datasets and compares emerging behavioral risk structures conceptually rather than computationally. As shown in Figure 1, the methodology consists of five phases: data acquisition, data preprocessing, statistical analysis, modeling based on machine learning, and evaluation.

### 3.1 Data acquisition

This study utilizes data from three primary sources: NHANES merged dataset, Kaggle Social Media dataset, and a Personalized Survey dataset. These datasets provide a comprehensive foundation for analysing the impact of substance use, social media engagement, and associated psychological factors.

#### 3.1.1 National Health and Nutrition Examination Survey (NHANES)

NHANES data were obtained from the National Center for Health Statistics (NCHS), Centers for Disease Control and Prevention (CDC), via the publicly accessible NHANES repository. De-identified participant-level datasets were downloaded in XPT format and merged across demographic, alcohol use, tobacco use, and sleep modules using the respondent identifier (SEQN). No administrative approval was required for access. The NHANES merged dataset represents a large, nationally representative sample of the civilian, non-institutionalized population of the United States, collected using a multistage, stratified probability sampling design. Participants span a broad range of ages, genders, educational levels, marital statuses, and socioeconomic backgrounds, enabling population-level inference regarding health behaviors. The dataset includes both younger and older adults, with balanced representation across gender categories, and captures variation in educational attainment and household characteristics.

#### 3.1.2 Social media influence data from Kaggle

The social media psychology dataset was obtained from the Kaggle data repository, where it is publicly hosted for research use. The dataset was accessed in CSV format and includes anonymized survey-based measures of digital engagement and psychological attributes. No restricted permissions were required. The dataset consist of people’s usage of WhatsApp, Facebook, Instagram, TikTok, and measures how daily hours spent on social media correlate with time spent elsewhere. The dataset includes platform usage frequency, daily time spent on social media, and self-reported psychological indicators, which were used as predictors in statistical and machine learning analyses. Perceived anxiety and perceived loneliness were included so that the emotional and psychological effects of social media use could be understood. These are critical components in trying to ascertain the relationship, if any, between how a person engages with other social media users and an addictive pattern as related to mental health. Although not designed to be nationally representative, the dataset captures meaningful variability in digital engagement intensity and psychosocial responses, making it suitable for exploratory and predictive behavioral analysis.

#### 3.1.3 Personalized survey dataset

Apart from the above two datasets, some personalized surveys were conducted to understand the addiction behaviors. The personalized survey dataset was collected from 236 participants representing diverse educational and occupational backgrounds using an online questionnaire platform. Participation was limited to adults, responses were anonymous, and no personally identifiable information was collected. This survey collects data regarding substance use, such as alcohol and nicotine consumption, as well as the mental impact of social media use. This included consideration of variables such as anxiety, stress, and loneliness as they were deemed useful in understanding the sort of emotions likely to be faced by potential subjects of addiction. The personalized responses, together with the wider datasets, would enable the research to carry out a holistic analysis of different patterns of addiction regarding the interplay of multiple factors and behaviors. The survey was administered anonymously, restricted to adult participants, and designed to collect non-identifiable behavioral and psychological responses for research purposes only. The survey collected self-reported measures related to substance use behaviors, digital engagement, and psychological well-being. Mental health related parameters, including perceived stress, emotional distress, anxiety, depressive symptoms, and social isolation were assessed using structured self-report items adapted from commonly used screening concepts rather than full clinical diagnostic instruments. Respondents rated their experiences using ordinal Likert-type scales reflecting frequency or intensity (e.g., low to high, never to very often). These measures were intended to provide contextual insight into behavioral and emotional patterns and were not used for diagnostic classification or primary model training.

To systematically analyze behavioral, psychological, and digital engagement factors, variables from all three datasets were organized into structured categories. Table 1 presentes the complete set of attributes extracted from the NHANES, Kaggle Social Media, and Personalized Survey datasets. These attribute groupings enabled consistent preprocessing, facilitated targeted statistical analysis, and supported dataset specific machine learning modeling. NHANES variables contributed demographic and lifestyle indicators, the Kaggle dataset supplied digital behavioral and psychometric measures, and the personalized survey provided context sensitive insights into self-reported mental health, environmental influences, and substance use experiences. The personalized survey was not used for primary statistical inference or model training, but to contextualize and interpret patterns observed in the large-scale secondary datasets.

**Table 1:** Consolidated summary of attributes across NHANES, Kaggle social media, and personalized survey datasets.

Category	Variable codes & descriptions
Social media influence data (NHANES)	
Demographic variables	RIAGENDR (Gender); RIDAGEYR (Age at screening); DMDEDUC2 (Education level – adults 20+); DMDMARTZ (Marital status); DMDHREDZ (Household reference person’s education level)
Alcohol consumption patterns	ALQ111 (Ever consumed alcohol); ALQ121 (Consumption frequency – last 12 months); ALQ130 (Avg. drinks per day – last 12 months); ALQ142 (Days with 4/5 + drinks – past year); ALQ270 (Times consuming 4/5 drinks within 2 h – past year); ALQ280 (Times consuming 8 + drinks – past year); ALQ151 (Ever consumed 4/5 + drinks daily); ALQ170 (4/5 drinks occasions – past month)
Tobacco and nicotine use	SMQ681 (Smoked tobacco – last 5 days); SMQ846 (Used e-cigarettes – last 5 days); SMQ851 (Used smokeless tobacco – last 5 days); SMQ863 (Used nicotine replacement – last 5 days); SMDANY (Used any tobacco – last 5 days); SMQ020 (Smoked 100 + cigarettes lifetime); SMQ040 (Current smoking status)
Smoking behavior & history	SMD641 (Days smoked – past 30 days); SMD650 (Avg. cigarettes/day – past 30 days); SMD100MN (Cigarette menthol indicator); SMQ621 (Total cigarettes smoked lifetime); SMD630 (Age first smoked a whole cigarette)
Sleep patterns	SLD012 (Sleep hours weekdays/workdays); SLD013 (Sleep hours weekends)
National Health and Nutrition Examination Survey	
Demographics and social media use	Sex; Age; School Failure; Type of Social Media Used (WhatsApp, Facebook, Instagram, Twitter, Snapchat, TikTok, Other); Time Spent on Social Media; Daily Use of Social Media; Primary Device Used for Social Media
Psychological and behavioral aspects	Time Perception during Social Media Use; Perceived Anxiety during Social Media Use; Perceived Loneliness; Frequency of Social Media Checking; Engagement in Video Games and Online Play; Time Spent Playing Video Games Online; BSMAS (Bergen Social Media Addiction Scale); BSMAS Cut-Off Score; RSES (Rosenberg Self-Esteem Scale); CSIQ-A (Cognitive Social Interaction Questionnaire – Adolescent); STAI Y-1 (State Anxiety Inventory – Short Form); STAI Y-2 (Trait Anxiety Inventory – Short Form)
Personalized survey variables	
Demographics and background info	Age; Gender; Education Level; Occupation
Substance use, history & influences	Substance Use History; Initial Use Reason (peer pressure, stress, curiosity, etc.); Recent Substance Use; Substance Use Frequency; Substance Dependence; Impact of Substance Use on Mental Health; Professional Help Sought for Substance Use
Mental health and psychological factors	Stress Levels; Emotional Distress; Anxiety; Depression; Social Isolation; Family and Peer Influence on Substance Use; Media and Societal Influence on Substance Use
Community and prevention strategies	Community Acceptance of Substance Use; Perceived Availability of Support Resources; Community-Based Prevention Suggestions

A comprehensive descriptive analysis was conducted to establish baseline prevalence rates and behavioral distributions across both cohorts, as detailed in Table 2. In this context, Mean and Standard Deviation (SD) are utilized for continuous variables to describe the mathematical average and the degree of variance within the sample. Conversely, Prevalence is employed for categorical variables to identify the mode (the most frequent value) and its relative percentage within the total sample. The demographic and behavioral profile of the study population reveals distinct patterns of stability contrasted with health-related vulnerabilities. The NHANES dataset is predominantly middle aged (Mean = 53.57, SD = 18.06) with consistent sleep patterns averaging approximately 7.7 to 8.3 h. Behavioral data indicates significant substance exposure, with 69.5% of participants having consumed alcohol; however, a bifurcated usage pattern is evident, as 48.4% reported no consumption within the past year. In the digital domain, while primary device usage is highly uniform (91.1%), psychological markers suggest an elevated baseline for trait anxiety (STAI Y-2 Mean = 46.93) and moderate levels of social media addiction (BSMAS Mean = 13.76). These details provide the essential baseline for the subsequent risk stratification, which identifies 32.6% of the digital cohort as falling within the high-behavioral-risk category. Primary variables included substance use indicators, psychological scale scores, and digital engagement measures. Secondary variables comprised demographic attributes such as age, gender, and education level, which were used for stratification and contextual interpretation.

**Table 2:** Descriptive analysis and population characteristics.

Category	Variable code	Description	Statistics
Demographics	RIAGENDR	Gender	Prevalence: 2.0 (55.3%)
	RIDAGEYR	Age	Mean = 53.57, SD = 18.06
	DMDEDUC2	Education Level	Prevalence: 5.0 (36.7%)
	DMDMARTZ	Marital Status	Prevalence: 1.0 (56.2%)
	DMDHREDZ	Household Ed	Prevalence: 2.0 (98.1%)
Alcohol	ALQ111	Ever Drank	Prevalence: 1.0 (69.5%)
	ALQ121	Frequency	Prevalence: 0.0 (48.4%)
	ALQ130	Avg Drinks/Day	Mean = 1.27, SD = 2.03
	ALQ142	Binge Days	Prevalence: 0.0 (74.4%)
	ALQ270	Binge 2 h	Prevalence: 0.0 (87.0%)
	ALQ280	8 + Drinks	Prevalence: 0.0 (90.3%)
	ALQ151	Binge Ever	Prevalence: 2.0 (55.1%)
	ALQ170	Binge Month	Mean = 0.63, SD = 3.06
Tobacco	SMQ681	Tobacco 5d	Prevalence: 2.0 (68.9%)
	SMQ846	E-Cigs	Prevalence: 2.0 (77.5%)
	SMQ851	Smokeless	Prevalence: 2.0 (80.5%)
	SMQ863	Nicotine Replace	Prevalence: 2.0 (80.5%)
	SMDANY	Any Tobacco	Prevalence: 2.0 (64.8%)
	SMQ020	100 + Cigs	Prevalence: 2.0 (60.9%)
	SMQ040	Current Smoking	Prevalence: 2.3395004625346902 (60.9%)
	SMD641	Days Smoked	Mean = 12.73, SD = 1.30
	SMD650	Avg Cigs/Day	Mean = 18.09, SD = 4.28
	SMD100MN	Menthol	Prevalence: 0.414468085106383 (86.2%)
Sleep	SLD012	Sleep Weekday	Mean = 7.70, SD = 1.82
	SLD013	Sleep Weekend	Mean = 8.33, SD = 1.97
Digital behavior	Sex	Sex	Prevalence: 2 (57.8%)
	Age	Age	Prevalence: 18 (23.3%)
	School failure	School Failure	Prevalence: 0 (64.7%)
	Time spent on social media	Usage Time	Prevalence: 1 (38.4%)
	Daily use of social media	Daily Freq	Prevalence: 1 (91.9%)
	Which device	Primary Device	Prevalence: 0 (91.1%)
	Time’s flow	Time Perception	Prevalence: 1 (49.2%)
	Perceived anxiety during SM use	SM Anxiety	Prevalence: 1 (82.9%)
	SM checking	Checking Freq	Prevalence: 1 (55.0%)
	Video-games and online play	Gaming Status	Prevalence: 1 (26.0%)
	Time spent on line playng videogames	Gaming Time	Prevalence: 0 (31.0%)
Psychological	BSMAS	Addiction Scale	Mean = 13.76, SD = 4.41
	RSES	Self-Esteem	Mean = 17.07, SD = 4.71
	CSIQ-A	Social Interaction	Mean = 21.35, SD = 5.15
	STAI Y-2	Trait Anxiety	Mean = 46.93, SD = 5.52

### 3.2 Key terms and definitions

- Digital well-being refers to an individual’s perceived psychological and emotional state associated with interaction with digital platforms, including experiences of anxiety, loneliness, self-esteem, and self-regulation during digital engagement.
- Digital stress denotes perceived emotional strain or cognitive burden associated with digital interactions, such as compulsive checking, information overload, social comparison, or prolonged screen exposure.
- Digital engagement refers to the extent and pattern of interaction with digital platforms, including time spent on social media, frequency of checking, platform diversity, and participation in online activities such as video gaming or content creation.
- Social media addiction is defined as problematic or compulsive use of social media platforms characterized by loss of control, preoccupation, and negative psychological impact.
- Elevated behavioral risk refers to a relative risk profile inferred from combinations of demographic, psychological, behavioral, and digital engagement features within a dataset. It does not imply clinical diagnosis but indicates similarity to patterns historically associated with higher substance use intensity or psychological vulnerability.
- Psychological vulnerability describes susceptibility to adverse emotional or behavioral outcomes associated with elevated anxiety, low self-esteem, loneliness, or maladaptive coping behaviors. These constructs are measured using standardized self-report scales.
- Substance-use indicators include behavioral and self-reported measures related to alcohol and tobacco consumption, such as frequency, intensity, binge episodes, and dependence-related items, as available in each dataset.
- Risk profile refers to the multivariate pattern of predictor variables that collectively characterize an individual’s relative position along a substance use or psychological vulnerability continuum within a dataset.
- Triangulation of evidence refers to the comparative interpretation of findings across independent datasets to identify consistent or divergent behavioral patterns without individual-level data integration.

### 3.3 Data cleaning pre processing

Attributes with more than 30% missing of the total data, were excluded from the analysis. For numerical attributes with missing data <30% were imputed with the median of the column. For categorical variables, such as gender, education level, and marital status, missing data were imputed with the mode. Normalizing and standardizing datasets should be conducted before any analysis, to ensure that no numerical attributes of different scales influence the outcome. Alcohol consumption (ALQ130, ALQ270, ALQ280) and Tobacco use (SMD650, SMD641) were normalized with Min-Max Normalization. Normalization through Min-Max scaling works well only for uniformly distributed data. Unfortunately, some variables like Self-reported anxiety levels and Time spent on social media are highly skewed. So these attributes are normalized with Z-score standardization, which transforms the data to follow a standard normal distribution (mean = 0, standard deviation = 1).

### 3.4 Population characteristics

Following the data cleaning and preprocessing stages, a descriptive analysis was conducted to establish foundational prevalence rates and behavioral distributions across the two datasets. In the NHANES dataset (*N* = 9,232), the results indicate a significant subset of abstainers, with 48.4% of the sample reporting no alcohol consumption in the past year (ALQ121). However, a distinct cluster of high-intensity use was identified: 14.2% of participants are categorized as frequent drinkers (consuming alcohol three or more times per week), and 14.1% meet the criteria for binge-drinking prevalence (ALQ151). Active tobacco uses or significant recent exposure (SMDANY) was observed in 16.7% of the population. The average age of this cohort is 53.85 years (SD = 18.14), providing a statistically diverse foundation for predictive models.

In the Social Media dataset, the average daily usage was recorded at 1.71 h (SD = 1.20). Psychological assessments reveal a cohort with elevated baseline levels of trait anxiety, with a mean STAI Y-2 score of 47.05 (SD = 5.81), and moderate self-esteem markers (RSES mean = 16.59, SD = 5.01). The Bergen Social Media Addiction Scale (BSMAS) showed a mean score of 13.07 (SD = 4.48). To provide the required stratification for behavioral risk, the population was partitioned based on the binned distribution of these addiction markers, revealing an actual prevalence of 39.9% Low Risk, 27.5% Moderate Risk, and 32.6% High Risk individuals. These benchmarks substantiate the classification performance reported hereafter, demonstrating that the models effectively learn from a representative spread of digital and psychological patterns.

### 3.5 Statistical techniques

We performed statistical analysis to understand the relationships between demographic characteristics, psychological states, digital-engagement behaviors, and indicators of substance use in the three datasets. As NHANES, Kaggle social media, and Personalized Survey datasets differ substantially in structure and measurement scales, each dataset was analyzed independently. The statistical procedures primarily used descriptive assessments and correlation based techniques, emulating the empirical results detailed in the results section. Descriptive statistics first summarize the distribution of major behavioral variables for each dataset. These provide a first foray into understanding how patterns of substance use differ in comparison to digital behaviors across demographic divisions. For instance, NHANES examples show age trends in consumption for alcohol and for nicotine, while intensity and psychological variables related to anxiety and loneliness are brought as examples from the Kaggle data about the social-media variable. Stress, emotional distress, and perceptions of community, under similar themes, were observable in the personalized survey captured. These trends were also visualized in histograms, bar charts, and scatter plots to find some more potentially interesting variables for the modeling work. Correlation analysis was also the main inferential tool as it corresponds directly to the multivariate results reported. Two types of correlation coefficients were computed depending on variable properties. Pearson’s correlation coefficient was applied to assess linear relationships between continuous variables, such as the association between alcohol consumption and age or between daily digital engagement and social-media addiction scores. The Pearson coefficient *r* is defined in Equation 1.


$$r=\frac{\sum_{i=1}^{n}(x_{i}-\bar{x})(y_{i}-\bar{y})}{\sqrt{\sum_{i=1}^{n}{\left(x_{i}-\bar{x}\right)}^{2}\sum_{i=1}^{n}{\left(y_{i}-\bar{y}\right)}^{2}}}$$ (1)

For variables that were ordinal or exhibited skewed distributions, Spearman’s rank correlation coefficient ρ was used. This method captures monotonic relationships, such as associations among perceived loneliness, anxiety scores, or frequency of checking social media. Spearman’s coefficient is given in Equation 2.


$$\rho =1-\frac{6\sum d_{i}^{2}}{n(n^{2}-1)}$$ (2)

where $d_{i}$ represents the ranked difference between paired observations.

To identify in what relationships factors of substance usage and digital behavior level outcomes are associated or predictively important, correlation heatmaps were generated over both NHANES and the Kaggle dataset. These correlation structures directly served to inform feature selection into the models that machine learning will run, thus preserving only the most relevant predictors, whilst actually shedding redundancy that would otherwise be present. To a large extent the analysis of the datasets occurred separately, but the insights statistically displayed from each were brought together conceptually for broad behavioral patterning. The NHANES results indicated that young adults were more likely to consume alcohol in greater amounts, while Kaggle data indicated that excessive engagement in the digital world was associated with angst and loneliness. The personalized survey lent self-reference to these findings; with respondents often citing stress, peer pressure, and digital fatigue adding to substance use and social media problematic behaviors. The cross-pollinating insights inform the philosophical holistic conception without any need for statistical integration of the datasets. Finally, all the statistical analyses would feed very importantly into the modeling predictive pipeline. Thus, edifying have the correlation findings in refining the feature sets and guiding the interpretation of model outputs through validating associations highlighted through machine-learning feature importance. In this way, the statistical layer sets a solid foundation for rigorous understanding of how the interaction between demographic variables, psychological attributes, and digital engagements defines the consumption pattern.

Potential sources of bias include self-reporting bias in social media and survey datasets, platform-specific sampling bias, and demographic imbalance. These were mitigated by analyzing datasets independently, using robust descriptive and correlation-based methods, and interpreting findings comparatively rather than inferentially across datasets. Model performance was evaluated using multiple metrics to reduce reliance on single-measure conclusions.

### 3.6 Machine learning models

Machine learning models were applied on NHANES and Social Media datasets to predict behavioral risk categories and estimate continuous indicators of substance use and psychological states. Individuals classified as “higher risk” represent those whose multivariate behavioral patterns resemble higher substance use intensity within the same dataset. Risk categorization was dataset-specific and does not constitute a standardized or transferable risk score. Since each dataset is different in structure, scale, and target variables, the models were trained separately on each source, preserving their contextual validity and ensuring that performance metrics reflect dataset-specific behavioral patterns. The modeling framework encompasses classification and regression approaches, which allows for a thorough analysis of both categorical and continuous behavioral outcomes. Five classification algorithms: Logistic Regression, Support Vector Regression (in classification mode), Random Forest, AdaBoost, and XGBoost were implemented. Logistic Regression was intended to serve as a linear baseline model against which predictive performance could be benchmarked, encoding into a viewpoint interpretable relationships between predictors and risk categories. Random Forest, AdaBoost, and XGBoost applies ensemble approaches that are still considered to be able to capture nonlinear interactions between demographic, psychological, and digital-engagement variables. Whereas, XGBoost would be naturally applicable to high-dimensional behavioral data due to its gradient boosting capability with built-in regularization. SVR was classified by mapping its continuous output to discrete class labels, allowing for nonlinear decision boundaries, maintaining, however, a simple model.

For classification of participants into risk categories of substance use or digital engagement from each dataset, accuracy and F1-score were used to assess model performance, yielding a balanced evaluation in moderate class-imbalance situations. For hyperparameter optimization, a grid search was performed on all classifiers, varying parameters like the number of estimators, learning rate, kernel setup, and maximum depth of tree. This tuning procedure gave a significant boost in predictive ability by improving performance across the two datasets, with model evaluation results confirming such success. There were two basic regression models that were implemented for continuous behavioral outputs like levels of alcohol consumption, intensity of nicotine use, scores on psychotherapy scales, and daily usage of social media: Linear Regression and Decision Tree Regression. Linear Regression provided an explanatory view as a baseline for modeling additive relationships between predictors. Decision Tree Regressor captures nonlinear dependence and interaction effects. The performance of these models was calculated using Mean Squared Error (MSE), Mean Absolute Error (MAE), and R^2^. The regression results elucidated how far demographic, psychological, and digital-behavior variables could account for continuous variations in substance use behaviors.

This study proposed a modular and replicable analytical framework rather than a single integrated predictive system. The framework was designed to be applied to heterogeneous behavioral datasets that differ in structure, scale, and sampling context. Each dataset is processed independently using a standardized pipeline comprising (i) dataset-specific preprocessing, (ii) descriptive and correlation-based statistical analysis, (iii) machine learning–based risk modeling, and (iv) comparative interpretation across data sources. The inputs to the framework consist of demographic attributes, behavioral indicators, psychological scales, and digital engagement measures available within a given dataset. The processing stages include feature normalization, correlation analysis, model training with hyperparameter tuning, and performance evaluation using dataset-appropriate metrics. The outputs are relative behavioral risk profiles, key predictive indicators, and interpretable patterns that can be compared across populations and contexts. Rather than merging datasets at the individual level, the framework emphasizes triangulation of evidence across independent data sources. This design supports consistent application of the analytical pipeline across heterogeneous datasets.

## 4 Experimental results and discussion

This section reports results obtained from independent analyses of the NHANES dataset, a social media psychology dataset, and a personalized survey, following the analytical pipeline described in the Methodology. Results are presented in a structured progression, beginning with identification of the most influential predictors through feature-importance analysis, followed by examination of correlation patterns and group-level statistical variation, and concluding with evaluation of machine-learning model performance. This organization enables a systematic interpretation of how demographic characteristics, psychological states, and digital engagement behaviors collectively shape substance use risk and mental well-being across heterogeneous data sources.

The NHANES dataset represents a nationally representative adult population with broad variability in age, gender, educational attainment, and lifestyle behaviors. Alcohol and tobacco consumption variables exhibited right-skewed distributions, with a majority of respondents reporting low to moderate use and a smaller proportion reporting heavy or binge consumption patterns. The social media psychology dataset primarily comprised adolescents and young adults, reflecting populations with high levels of digital engagement. Considerable variability was observed in daily social media usage time, frequency of checking behaviors, and gaming activity. Psychological measures, including anxiety, loneliness, self-esteem, and social media addiction scores, demonstrated heterogeneous distributions, indicating the presence of both low-risk and high-risk behavioral profiles. The personalized survey dataset (*N* = 236) consisted of a heterogeneous adult sample spanning diverse educational and occupational backgrounds. Respondents reported varying levels of substance use, perceived digital stress, and psychological well-being. Although not used for primary statistical inference or model training, this dataset provided contextual insight into subjective experiences and coping behaviors that complemented the large-scale secondary datasets. Gender was included as a demographic variable in both the NHANES and personalized survey datasets. Descriptive assessment indicated observable differences in substance use patterns across gender groups, consistent with established epidemiological evidence. While gender was not the primary focus of predictive modeling in this study, its inclusion provided important demographic context for interpreting behavioral risk patterns. Gender-specific stratified analysis was beyond the scope of the present study, which prioritized cross-domain behavioral and digital predictors, but remains an important direction for future investigation.

### 4.1 Feature importance and predictive indicators

**Figure 2:**
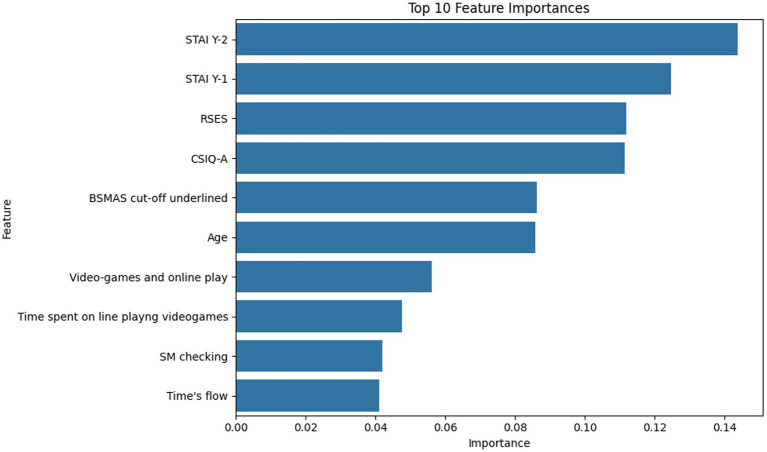
Feature importance in substance use analysis.

**Figure 3:**
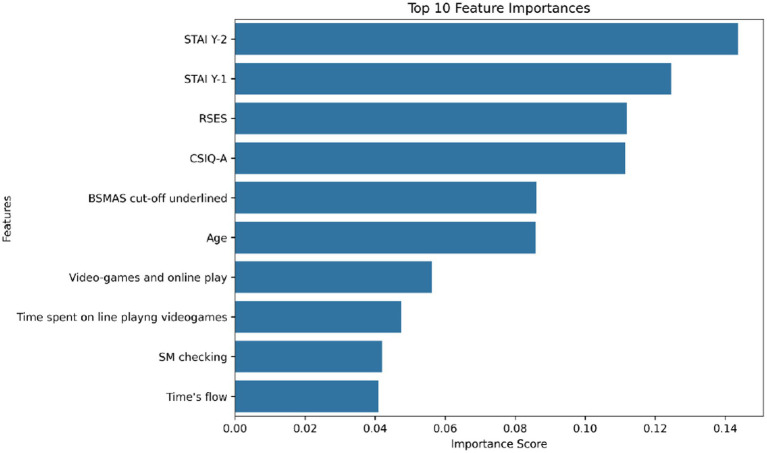
Feature importance in social media and psychological impact.

Across both datasets, the most influential predictors reflect a mixture of demographic attributes, substance use indicators, psychological scales, and digital behavior variables. As shown in the Figure 2, the most significant predictive variable in the NHANES dataset is the frequency of alcohol consumption (ALQ121), followed by age (RIDAGEYR) and key indicators of dependency and binge behavior, such as ALQ151 and ALQ142. These findings align with established epidemiological patterns where younger populations and binge-drinking habits serve as strong statistical signals for elevated substance use risk. For the social media dataset, as illustrated in the modified Figure 3, the predictive landscape is dominated by psychological and digital engagement variables. Trait anxiety (STAI Y-2) and state anxiety (STAI Y-1) emerged as the highest-ranked psychological predictors, indicating that both chronic and transient emotional states are critical markers of digital engagement and subsequent substance use tendencies. Self-esteem (RSES) and the Bergen Social Media Addiction Scale (BSMAS) cutoff scores also hold significant predictive weight, while engagement frequency and gaming intensity are deemed moderately important. Importantly, these feature importance rankings, detailed in Table 3, reflect the predictive contribution within the machine-learning framework rather than deterministic causal relationships. While these attributes effectively identify individuals within elevated risk profiles, they do not imply a direct causal mechanism or clinical diagnosis.

**Table 3:** Top features in substance use and social media influence analysis.

Feature	Category	Importance	Reason for high importance
ALQ121	Alcohol consumption	Highest	Strongest predictor of substance uses behaviors.
RIDAGEYR	Age	High	Age significantly influences drinking and smoking habits.
ALQ151	Alcohol dependency	High	Measures alcohol dependence, affecting substance use behaviors.
ALQ142	Alcohol binge frequency	High	Frequent binge drinking is a key indicator of substance abuse.
STAI Y-2	Trait anxiety	Highest	Strongly related to long-term psychological distress.
STAI Y-1	State anxiety	High	Anxiety fluctuations impact social media engagement and substance use.
RSES	Self-esteem	High	Low self-esteem correlates with anxiety and risky behaviors.
BSMAS cut-off score	Social media addiction	High	Indicates problematic social media use and its psychological effects.
SM checking	Social media engagement	Medium	Frequent checking is linked to anxiety and mental health concerns.
Video-games & online play	Digital entertainment	Medium	Excessive screen time influences mental health and behaviors.
Time spent on social media	Social media usage	Medium	High engagement correlates with anxiety and self-perception issues.

### 4.2 Correlation patterns between digital behavior and substance use

**Figure 4:**
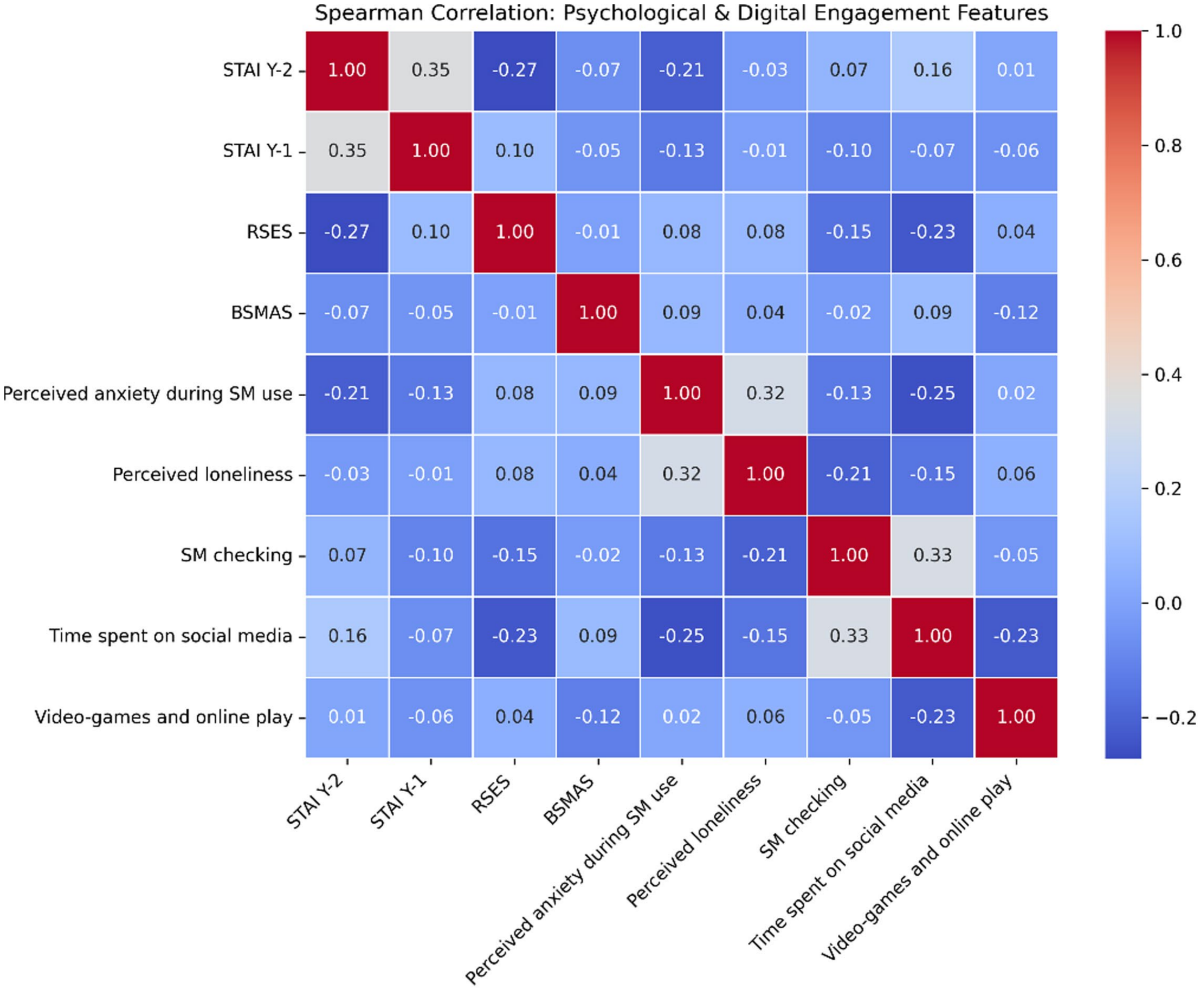
Correlation heatmap for social media and mental health.

**Figure 5:**
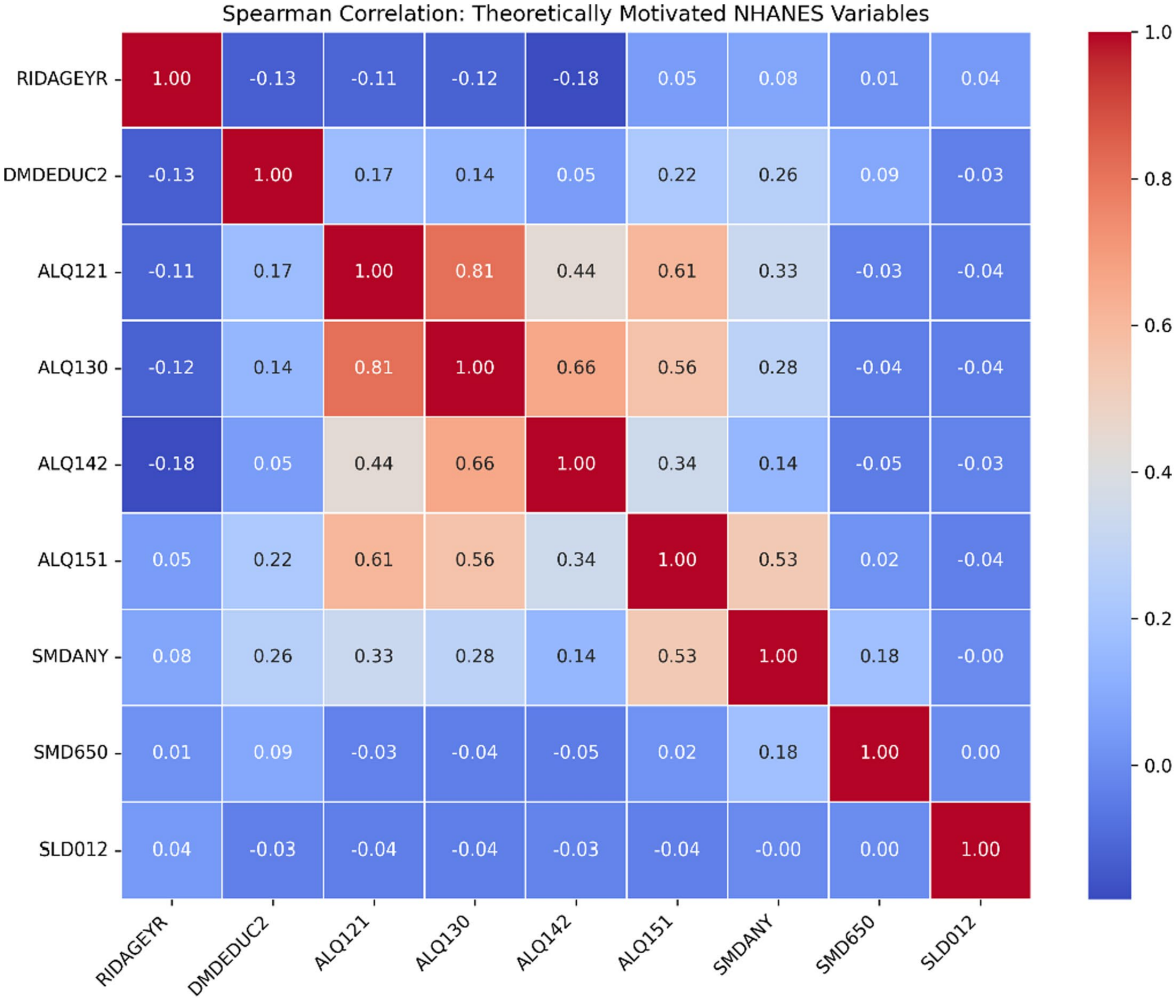
Correlation heatmap for NHANES.

The correlation analysis, refined through Spearman’s rank coefficients to account for ordinal scales and skewed distributions, reveals nuanced, non-linear associations across both datasets. As shown in Figure 4, psychological indicators in the social media dataset, such as trait anxiety (STAI Y-2) and state anxiety (STAI Y-1), exhibit a moderate positive correlation (0.35), confirming their internal consistency as indicators of psychological distress. Interestingly, perceived anxiety during SM use shows a positive relationship with perceived loneliness (0.32), yet both demonstrate weak or inverse relationships with total time spent on social media (−0.25 and −0.15 respectively). This suggests that anxiety levels may plateau or stabilize regardless of usage duration, supporting the theory that qualitative engagement factors and adaptive coping mechanisms moderate outcomes rather than absolute screen time. Furthermore, compulsive behaviors like SM checking frequency show a strong positive association with time spent on social media (0.33), while intensive video-games and online play correlates inversely with total social media duration (−0.23), reinforcing the distinct nature of digital engagement across platforms. In the NHANES dataset, the filtered analysis presented in Figure 5 highlights key demographic and lifestyle drivers. Age (RIDAGEYR) maintains a consistent inverse relationship with alcohol consumption frequency (ALQ121, −0.11) and average drinks (ALQ130, −0.12), confirming higher usage frequency among younger populations. Conversely, educational attainment (DMDEDUC2) shows a modest positive association with alcohol consumption indicators (e.g., 0.26 with SMDANY and 0.17 with ALQ121), likely reflecting social-contextual influences within higher education environments rather than academic stress. Predictably, strong correlations are observed between internal substance use metrics, such as ALQ121 and ALQ130 (0.81), validating the reliability of these self-reported behavioral clusters. Collectively, these findings as visualized in Figures 4, 5 demonstrate that substance use and digital engagement are governed by context-dependent, bidirectional mechanisms, justifying a move away from simplified linear risk models toward the integrated, behavioral fingerprinting framework proposed in this study.

It is important to note that correlation coefficients were interpreted primarily in terms of their direction and relative magnitude rather than statistical detectability. Given the large sample size of NHANES, small correlation values (e.g., |r| ≈ 0.10–0.15) may be observable but represent weak effects with limited substantive relevance. Accordingly, such associations were not treated as meaningful drivers of behavior in isolation but were considered only as part of broader multivariate patterns. The analysis emphasizes practical interpretation of effect size over nominal statistical sensitivity.

### 4.3 Statistical and group-level variation

It was a very complex relationship that is found in Table 4, demographic, psychological, and behavioral factors influencing substance use and social media engagement together. Age was an important factor; for young people, peer pressure tended to lead to substance use, while older people indicated that they feel lonelier, which possibly leads them to turn to other means for coping. Notably, higher education associated with increased alcohol consumption was likely the result of the social environment, but not the pressure from the school itself to perform. Altogether, these will imply that with traditional assumptions regarding substance use, maybe not everything has been captured in the real, nuanced reality of the individual and the social factors that shape it. Psychological well-being is a major behavioral associated with substance usage, with both anxiety and loneliness emerging as important constructs in this regard.

**Table 4:** Model performance before and after hyperparameter tuning.

Model	Before hyperparameter tuning				After hyperparameter tuning			
	NHANES accuracy	NHANES F1 score	Social media accuracy	Social media F1 score	NHANES accuracy	NHANES F1 score	Social media accuracy	Social media F1 score
Random Forest	0.7513	0.7995	0.659	0.650	0.724	0.714	0.865	0.822
XGBoost	0.7535	0.8036	0.651	0.641	0.718	0.710	0.827	0.791
AdaBoost	0.7546	0.7533	0.624	0.608	0.741	0.718	0.827	0.749
SVR	0.2099	0.2029	0.637	0.621	0.733	0.699	0.827	0.749
Logistic regression	0.4284	0.4242	0.6484	0.631	0.710	0.670	0.830	0.753

### 4.4 Machine learning model performance

In this study, elevated behavioral risk does not refer to a clinical diagnosis or deterministic outcome. Rather, it denotes a relative risk profile inferred from the joint distribution of demographic, behavioral, psychological, and digital engagement features within each dataset. Individuals classified as higher risk represent those whose combined feature patterns resemble profiles historically associated with greater substance use intensity, dependency indicators, or psychological vulnerability, as defined by the target labels available in each dataset. The predictive performances of various models on both NHANES and Social Media datasets before and after hyperparameter tuning were evaluated and the results is shown in Table 5. All classification models operated on multivariate feature spaces, learning collective patterns across variables rather than evaluating individual predictors in isolation. Model evaluation was based on overall accuracy and F1-score to balance between precision and recall measures. The reported accuracy and F1-scores therefore indicate pattern recognition capability under observed conditions, not diagnostic certainty. Before tuning hyperparameters, the best performance was given by XGBoost in the NHANES dataset with accuracy of 0.7535 and F1 score of 0.8036. Random Forest performed exceptionally on the model with the Social Media dataset, providing accuracy of 0.6593 and an F1 score of 0.6501 for comparison with other algorithms. However, hyperparameter tuning truly gave the models a new shine. Of the improvements, Random Forest had the highest score for the Social Media dataset at an accuracy score of 0.865, coupled with an F1 score of 0.822. AdaBoost, on the other hand, did well in prediction based on the NHANES dataset, with an accuracy score of 0.741 and an F1 score of 0.718. These improvements show that the enhancements in predictive capabilities highly depend on employing specific hyperparameter tuning strategies, such as adjusting the learning rate, maximum depth, or number of estimators.

**Table 5:** Statistical analysis results and insights.

Category	Test type	Key findings	Interpretation	Correlation (Pearson, Spearman)
Education & substance use	Pearson/Spearman Correlation	Higher education correlated with increased alcohol/smoking	Social settings in higher education may influence substance use	DMDEDUC2 vs. ALQ111 (0.0908, 0.0958)
Age & substance use	Pearson/Spearman Correlation	Younger individuals consume more alcohol	Peer influence plays a role in substance use among youth	RIDAGEYR vs. ALQ130 (−0.1283, −0.1210)
Age & loneliness	Pearson/Spearman Correlation	Older individuals report higher loneliness	Loneliness can be a psychological trigger for substance use	Age vs. Perceived Loneliness (0.1297, 0.1401)
Anxiety & self-esteem	Pearson Correlation	Higher self-esteem linked to lower anxiety levels	Self-esteem might act as a protective factor	RSES vs. STAI Y-2 (−0.26)
Social media & psychological factors	Pearson/Spearman Correlation	More social media time correlated with less anxiety & loneliness	Online interactions provide emotional support	Time Spent on Social Media vs. Perceived Anxiety (−0.2559, −0.2533)

To explore relationships between predictors and continuous behavioral outcomes, regression models were applied using Mean Squared Error (MSE), Mean Absolute Error (MAE), and the coefficient of determination (R^2^) as evaluation metrics. On the NHANES dataset, Linear Regression and Decision Tree Regression achieved modest explanatory performance (R^2^ = 0.30 for both models), indicating that a limited but meaningful proportion of variance in substance use indicators could be accounted for by demographic and lifestyle variables (Table 6). In contrast, regression performance on the social media dataset was substantially weaker (Linear Regression R^2^ = 0.15; Decision Tree Regression R^2^ = −0.37), suggesting that simple regression models are insufficient to capture the highly nonlinear, context-dependent, and psychologically complex relationships inherent in digital-behavior variables. These results indicate limited generalization capacity for continuous outcome prediction in the social media dataset and underscore the exploratory nature of the regression analysis. Importantly, the purpose of these regression experiments was not to develop deployable predictive models, but to assess the extent to which linear and tree-based approaches can approximate behavioral trends within each dataset. The observed performance differences between NHANES and social media data highlight the greater structural regularity of traditional health variables compared with digital engagement measures, which likely require more advanced nonlinear or temporal modeling strategies. Accordingly, these regression results should be interpreted as hypothesis-generating rather than confirmatory, and as evidence of methodological limitations rather than predictive sufficiency. With these limitations mentioned, this study provides a realistic foundation for future AI-based risk assessment, highlighting that moving beyond simple linear models is a prerequisite for clinical-grade behavioral modeling.

**Table 6:** Regression model performance.

Model	NHANES (Before) MSE	NHANES (Before) MAE	NHANES (Before) R^2^	Social media (Before) MSE	Social media (Before) MAE	Social media (Before) R^2^
Linear regression	3.98	0.49	0.30	12.12	2.12	0.15
Decision tree regressor	4.81	0.37	0.30	21.54	2.65	−0.37

### 4.5 Interpretation of key findings and practical implications

This study reveals several findings that diverge from established narratives in digital psychology and substance use research. While previous researchers have frequently reported a linear relationship between social media use and adverse mental health outcomes, particularly anxiety and loneliness, suggesting that increased usage directly exacerbates psychological distress (4, 7, 13), the present study observed that anxiety levels remained relatively stable across varying degrees of social media engagement. This is quantitatively supported by the weak Spearman correlation coefficients visualized in Figure 4, which indicate that anxiety scores (STAI Y-1 and STAI Y-2) do not escalate linearly with usage duration. Similarly, loneliness did not uniformly increase with greater time spent online; instead, it fluctuated depending on the nature and context of engagement, challenging deterministic assumptions regarding digital media.

Volkow and Blanco (14) and Sun et al. (6) emphasized a reinforcing cycle whereby digital stress leads to increased social media use, often framed as maladaptive “doom-scrolling.” However, our analysis identified only a weak association between perceived stress and increased usage, suggesting that many individuals actively self-regulate digital behaviors during stressful periods. With respect to substance use, where Strickland and Smith (15) and Queen et al. (12) commonly linked greater exposure to increased alcohol and tobacco consumption, our findings reveal a nonlinear relationship. Moderate substance users exhibited higher digital engagement, while heavier users showed reduced activity, potentially due to functional impairment or social withdrawal.

Furthermore, while educational attainment is traditionally associated with lower social media usage as a protective factor (13, 16), this study found that highly educated individuals adopted moderated, purpose-driven patterns rather than disengagement. This aligns with the modest positive correlations in Figure 5 between education level (DMDEDUC2) and alcohol frequency (ALQ121), likely reflecting social-contextual influences. While gaming addiction is typically examined separately (11), we identified a strong association between intensive gaming and substance use. Additionally, binge drinking—traditionally attributed to peer influence (13, 14, 17)—was associated here with compulsive digital consumption. Unlike studies relying solely on self-reports (5, 18), we demonstrate the feasibility of machine-learning behavioral models to identify at-risk profiles. Finally, whereas prior research often ignored digital habits (19), our findings highlight digital stress as a salient factor in substance use vulnerability.

#### 4.5.1 Stable anxiety despite increased usage

Contrary to the widely cited linear dose response relationship between social media use and anxiety, anxiety scores in this cohort plateaued or even slightly declined at higher usage thresholds. This stabilization appears to stem from (a) algorithmic curation that keeps users within psychologically comfortable echo chambers, (b) active utilization of mental health oriented communities, and (c) self regulated avoidance of triggering content. The finding challenges blanket recommendations for screen-time reduction and suggests that platforms could be engineered to amplify protective content rather than merely restrict access.

#### 4.5.2 Self-regulation in response to digital stress

A substantial proportion of participants reported deliberately reducing social media engagement during periods of elevated stress, directly contradicting the “stress → doom-scrolling” pathway commonly described in the literature. This self-regulatory behavior highlights significant inter-individual differences in coping repertoires and underscores the limitation of universal digital-detox interventions. Personalized, adaptive strategies that teach mindful consumption and boundary setting are likely to be more effective than prescriptive time limits.

#### 4.5.3 Digital disengagement among heavy substance users

Heavy consumers of alcohol and cannabis exhibited markedly lower social media activity than moderate users. This pattern is consistent with progressive social withdrawal, shame-related avoidance, and cognitive/motivational deficits observed in advanced substance use disorders. Critically, it implies that passive monitoring of social media activity alone will fail to identify the highest-risk individuals, who may become digitally silent. Early-warning systems must therefore integrate offline behavioral markers or sudden drops in previously high digital activity as red flags.

#### 4.5.4 Purposeful engagement among highly educated users

Higher educational attainment did not reduce overall social media use but transformed its purpose from passive entertainment to professional networking, knowledge acquisition, and curated learning (e.g., LinkedIn, Reddit, Twitter academic communities). Greater digital literacy appears to enable self-regulation and value-aligned usage, reinforcing the importance of media-literacy programmes over restrictive policies.

#### 4.5.5 Primacy of engagement quality over quantity

Across all datasets, the emotional valence and intentionality of interaction proved far more predictive of mental health and substance use outcomes than absolute screen time. Active, meaningful engagement (e.g., participation in support groups, creation of content, or professional discourse) was associated with better psychological outcomes even at high usage levels, whereas passive consumption and toxic interactions were strongly detrimental. This finding represents a paradigm shift: digital well-being interventions should prioritize cultivating healthy engagement habits and digital literacy rather than focusing exclusively on duration-based restrictions. Taken together, insights derived independently from population-level health data, digital-behavioral datasets, and self-reported contextual evidence demonstrate that the relationship between digital environments and substance use is non-linear, highly individualized, and heavily moderated by the quality and purpose of engagement. They advocate for a move away from one-size-fits-all screen-time guidelines toward intelligent, behaviorally informed, and personalized prevention frameworks that harness the protective potential of digital ecosystems while mitigating their risks.

#### 4.5.6 Multi-source evidence synthesis for substance use risk assessment

Building on the analytical framework defined in the Methodology, this section synthesizes how findings from independent datasets collectively inform substance use risk interpretation. Although the three datasets were analyzed independently, their combined interpretation provides a multi-source evidence framework for understanding substance use risk. Each data source contributes a distinct but complementary perspective: NHANES captures population-level demographic and lifestyle correlates of substance use; the social media dataset reveals psychological and digital-behavioral risk signals that are not observable in traditional health surveys; and the personalized survey offers contextual insight into subjective experiences, coping mechanisms, and cultural influences. By triangulating these perspectives, the present study moves beyond single-source risk characterization. Patterns observed in one dataset are reinforced, contextualized, or bounded by findings from others, enabling a more nuanced interpretation of substance use vulnerability. This framework supports personalized risk profiling by integrating demographic susceptibility, psychological state, and digital engagement patterns, even in the absence of record-level dataset integration.

### 4.6 Practical implications for public health and clinical practice

Although the present study is exploratory and not intended for clinical deployment, several actionable implications emerge for public health practitioners and frontline clinicians. For public health practitioners, the findings suggest that substance use prevention strategies should move beyond uniform screen-time reduction policies. Programs may benefit from incorporating assessments of digital engagement quality, such as compulsive checking behavior, gaming intensity, and emotional responses to online interaction, rather than focusing solely on usage duration. Public health campaigns can leverage social media platforms as dual-purpose environments—both potential risk amplifiers and channels for supportive engagement by promoting digital literacy, self-regulation strategies, and constructive online participation rather than blanket avoidance. For clinicians and behavioral health professionals, the results highlight the value of incorporating brief, non-diagnostic screening questions related to digital stress, perceived loneliness, and compulsive online behaviors during routine substance use or mental-health assessments. These factors may serve as contextual indicators that help clinicians better understand coping patterns and psychosocial stressors associated with substance use vulnerability, even when traditional clinical indicators are absent. Importantly, the study does not advocate algorithm-driven clinical decision-making. Instead, the findings support the use of behavioral and digital-context indicators as adjunct information to inform risk awareness, patient counseling, and referral decisions. For policy makers and program designers, the results underscore the importance of culturally and contextually adaptive interventions. Prevention efforts should account for heterogeneity in digital behavior across age, education, and social context, recognizing that digital engagement may function as a coping mechanism for some individuals and a risk amplifier for others. Tailored interventions emphasizing healthy engagement patterns, rather than abstinence-based digital restrictions, may therefore be more effective at population scale.

## 5 Conclusion and future work

The present study has gathered the much-needed insight into the interrelationships existing within social media usage, mental health, substance use as well as behavioral inclinations like digital addiction. Population-level analysis of NHANES data demonstrated that traditional demographic and lifestyle factors remain foundational determinants of substance use risk, while analyses of social media and psychological datasets revealed how digital engagement and mental-health factors modulate and contextualize these baseline risks. The findings refute several generally accepted notions, especially those related to the linear correlation of social media with anxiety. Given the observed nonlinear relationship between digital engagement and mental health, prevention strategies should move away from simple screen-time limits. Instead, interventions should focus on the quality of digital interactions, targeting the specific behavioral patterns that occur before the observed anxiety plateau. This shifts the focus from the quantity of digital life to the nuances of self-regulation and individual differences. Unlike studies that rely exclusively on self-reported measures, this work adopts a predominantly data-driven approach using large-scale secondary datasets, complemented by a targeted personalized survey to provide contextual and interpretive insights. Ultimately, these findings emphasize that social media is both a risk factor and a potential tool for mental health support. This calls for a restructuring of paradigms in digital well-being strategies favoring content curation, digital literacy, and personalized support systems over blanket bans or restrictive usage limits.

The framework is intended for research and population-level behavioral surveillance rather than individual clinical decision-making. Its primary utility lies in identifying patterns that may inform future hypothesis-driven studies and prevention strategies. Given the exploratory nature of this study, formal multiple comparison correction and confidence interval estimation were not applied. Correlation coefficients were interpreted primarily in terms of direction and relative magnitude rather than statistical significance, and findings should be viewed as hypothesis-generating rather than confirmatory. The model training and evaluation were conducted within the same datasets and the present findings demonstrate internal predictive consistency rather than external generalizability. Future work should validate these patterns across independent cohorts to assess robustness and transferability and adopt a multi-dimensional dimension mechanism that would capture not only individual personalized behavioral patterns but also socio-cultural influences and shifts in the digital landscape. Another attractive area for future work is predictive modeling based on AI to improve supports for addiction and mental health. Findings show that not all people with substance use problems would be characterized on the social media spectrum as over-users or addicted. Future work should then check whether AI models could track online behaviors and recognize addiction or distress signals in real-time with personalized intervention based on the findings. This could enable the earliest identification of at-risk persons and thus enhance more effective support programs in the field of mental health. Comprehending these shifts can enable better development of effective digital literacy programs by policy makers and educators. Finally, research on nonlinear relationships between digital habits and well-being should be studied more in the future. Whereas stress drivers in certain individuals lead to overuse of social media, others exhibit self-regulation and controlled engagement.

## Data Availability

The raw data supporting the conclusions of this article will be made available by the authors, without undue reservation.

## References

[1] Amaro H Sanchez M Bautista T Cox R. Social vulnerabilities for substance use: stressors, socially toxic environments, and discrimination and racism. Neuropharmacology. (2021) 188:108518. doi: 10.1016/j.neuropharm.2021.108518, 33716076 PMC8126433

[2] Pomrenze MB Paliarin F Maiya R. Friend of the devil: negative social influences driving substance use disorders. Front Behav Neurosci. (2022) 16:836996. doi: 10.3389/fnbeh.2022.836996, 35221948 PMC8866771

[3] Belfiore CI Galofaro V Cotroneo D Lopis A Tringali I Denaro V . A multi-level analysis of biological, social, and psychological determinants of substance use disorder and co-occurring mental health outcomes. Psychoactives. (2024) 3:194–214. doi: 10.3390/psychoactives3020013

[4] Nawi AM Ismail R Ibrahim F . Risk and protective factors of drug abuse among adolescents: a systematic review. BMC Public Health. (2021) 21:2088. doi: 10.1186/s12889-021-11906-2 34774013 PMC8590764

[5] McKim TH Bauer DJ Boettiger CA. Addiction history associates with the propensity to form habits. J Cogn Neurosci. (2016) 28:1024–38. doi: 10.1162/jocn_a_00953, 26967944 PMC5046041

[6] Sun Y Liu Y Zhang J. Excessive enterprise social media use behavior at work: role of communication visibility and perspective of uses and gratifications theory. IEEE Access. (2020) 8:190989–1004. doi: 10.1109/ACCESS.2020.3032035

[7] Ahmed RR Qureshi JA Arshad F Hashem EAR Channar ZA Parmar V . The social media break-up: psycho-behavioral measures and implications. IEEE Access. (2022) 10:58116–35. doi: 10.1109/ACCESS.2022.3178839

[8] Woo J Kang SW Kim HK Park J. Contagion of cheating behaviors in online social networks. IEEE Access. (2018) 6:29098–108. doi: 10.1109/ACCESS.2018.2834220

[9] Tseng Y-L Su YK Chou WJ Miyakoshi M Tsai CS Li CJ . Neural network dynamics and brain oscillations underlying aberrant inhibitory control in internet addiction. IEEE Trans Neural Syst Rehabil Eng. (2024) 32:946–55. doi: 10.1109/TNSRE.2024.3363756, 38335078

[10] Martín CA Rivera DE Hekler EB Riley WT Buman MP Adams MA . Development of a control-oriented model of social cognitive theory for optimized mHealth behavioral interventions. IEEE Trans Control Syst Technol. (2020) 28:331–46. doi: 10.1109/TCST.2018.2873538 33746479, 33746479 PMC7977327

[11] Khalid MNA Iida H. Objectivity and subjectivity in games: understanding engagement and addiction mechanism. IEEE Access. (2021) 9:65187–205. doi: 10.1109/ACCESS.2021.3075954

[12] Queen O Jodoin V Pearcy LB Strickland WC. Agent-based dynamics of a SPAHR opioid model on social network structures (2022):2202.12261. doi: 10.48550/arXiv.2022.12261,

[13] Whitesell M Bachand A Peel J Brown M. Familial, social, and individual factors contributing to risk for adolescent substance use. J Addict. (2013) 2013:579310. doi: 10.1155/2013/579310, 24826363 PMC4008086

[14] Volkow ND Blanco C. Substance use disorders: a comprehensive update of classification, epidemiology, neurobiology, clinical aspects, treatment, and prevention. World Psychiatry. (2023) 22:203–29. doi: 10.1002/wps.21073, 37159360 PMC10168177

[15] Strickland JC Smith MA. The effects of social contact on drug use: behavioral mechanisms controlling drug intake. Exp Clin Psychopharmacol. (2014) 22:23–34. doi: 10.1037/a0034669, 24188170 PMC3926100

[16] Dogutas A. Sociological influences on addiction: culture and ethnicity. Open J Soc Sci. (2023) 11:617–29. doi: 10.4236/jss.2023.119038

[17] Awua J Tuliao AP Gabben-Mensah D Kanjor F Botor NJB Ohene L . Interpersonal communication and perceived norms as social influence mechanisms of e-cigarette use among adults: a systematic review. Am J Drug Alcohol Abuse. (2024) 50:291–304. doi: 10.1080/00952990.2024.2346928, 38832973 PMC12035739

[18] Shah-Mohammadi F. Finkelstein J. (2024). AI-powered social determinants of health extraction from patient records: A GPT-based investigation. 2024 IEEE First International Conference on Artificial Intelligence for Medicine, Health and Care (AIMHC), 109–112

[19] Ewald DR Strack RW Orsini MM. Rethinking addiction. Global Pediatric Health. (2019) 6:2333794X18821943. doi: 10.1177/2333794X18821943, 30719491 PMC6348542

[20] Jangra H. Shah R. Kumaraguru P. (2023). Effect of feedback on drug consumption disclosures on social media. Proceedings of the International AAAI Conference on Web and Social Media, 17, 435–446.

[21] Andy A. U. Guntuku S. (2020). Does social support (expressed in post titles) elicit comments in online substance use recovery forums?. 35–40.

[22] Bouzoubaa L. Aghakhani E. Song M. Trinh M. Rezapour R. (2024). Decoding the narratives: analyzing personal drug experiences shared on Reddit. 2406.12117.

[23] Houghton DC Spratt HM Keyser-Marcus L Bjork JM Neigh GN Cunningham KA . Behavioral and neurocognitive factors distinguishing post-traumatic stress comorbidity in substance use disorders. Transl Psychiatry. (2023) 13:296. doi: 10.1038/s41398-023-02591-3, 37709748 PMC10502088

[24] Tseng T.-J. Li Y.-M. (2024). A drug abuse detection based on user behavior and linguistic style. 2024 16th IIAI International Congress on Advanced Applied Informatics (IIAI-AAI), 127–130.

